# Apical Sparing of Longitudinal Strain and Myocardial Fibrosis in Hypertensive Patients and Spontaneously Hypertensive Rats: Based on Speckle Tracking and Histological Analysis

**DOI:** 10.1002/clc.70255

**Published:** 2026-01-15

**Authors:** Chunyan Huang, Yongxin Wu, Meiyan Lin, Yupeng Chen, Shengnan Lin, Liyun Fu, Huimei Huang

**Affiliations:** ^1^ Department of Ultrasound The First Affiliated Hospital of Fujian Medical University Fuzhou China; ^2^ Department of Ultrasound National Regional Medical Center, Binhai Campus of the First Affiliated Hospital, Fujian Medical University Fuzhou China; ^3^ Education Division The First Affiliated Hospital of Fujian Medical University Fuzhou China; ^4^ Department of Pathology The First Affiliated Hospital of Fujian Medical University Fuzhou China

**Keywords:** apical sparing, fibrosis, hypertension, longitudinal strain, systolic function

## Abstract

**Background:**

This study aimed to investigate regional myocardial strain and fibrosis distribution to analyze the apical sparing pattern and the relation with hypertrophy in hypertension.

**Methods:**

This study included clinical and experimental animal investigations. Seventy‐three hypertensive patients were divided into two groups: hypertension without left ventricular hypertrophy (HT‐NLVH) and hypertension with LVH (HT‐LVH). Six 16‐week‐old male spontaneously hypertensive rats (SHR) and six age‐matched male Wistar‐Kyoto (WKY) rats were included in this experiment. Echocardiographic measurements were obtained. Myocardial strain indexes, including global longitudinal strain (GLS), the basal, middle, and apical segmental LS (LS‐bas, LS‐mid, LS‐ap), and the proportion of LS‐ap/(LS‐bas + LS‐mid + LS‐ap) (P‐ap) were measured. The histological collagen volume fraction (CVF) and perivascular collagen area (PVCA) of basal and apical segments (CVF‐bas, CVF‐ap, PVCA‐bas, PVCA‐ap) were observed in all rats.

**Results:**

Despite preserved systolic function (FS, LVEF), the HT‐NLVH and HT‐LVH groups exhibited diastolic impairment (elevated LAVI, E/e’) (all *p* < 0.05). LS‐ap declined only in HT‐LVH, while LS‐mid and LS‐bas worsened from HT‐NLVH to HT‐LVH, and the HT‐LVH group exhibited a significantly elevated P‐ap (all *p* < 0.05). P‐ap was associated with LV remodeling indexes and E/e’ (all *p* < 0.05). Compared with WKY, LS‐bas decreased in SHR (*p* < 0.05). The SHR group demonstrated significantly elevated PVCA‐bas, PVCA‐ap, and CVF‐bas (*p* < 0.05), while the CVF‐ap had no significant difference.

**Conclusion:**

Myocardial dysfunction and fibrosis exhibited regional heterogeneity with predominant basal damage and apical sparing in hypertensive cardiac hypertrophy. This apical‐sparing pattern correlated significantly with both diastolic dysfunction and hypertrophic progression, suggesting its potential as a clinically observable hallmark.

## Graphical abstract

Hypertensive cardiac hypertrophy shows a “basal‐damage, apical‐sparing” pattern in strain and fibrosis. This apical sparing correlates with diastolic dysfunction and hypertrophy progression, highlighting its value as an observable clinical hallmark of adverse remodeling.

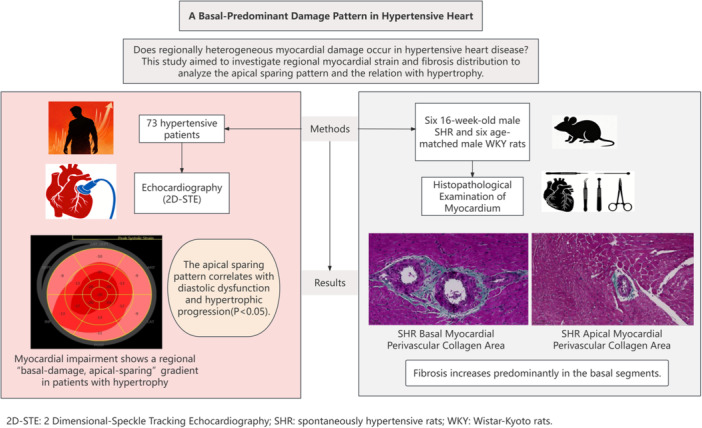

## 1 Introduction

Hypertension plays a critical role in the pathophysiology of cardiovascular disease [1]. Increased hemodynamic load in hypertension triggers adaptive myocardial hypertrophy, which progresses to structural remodeling, myocardial fibrosis, and eventual cardiac dysfunction [2]. Notably, hypertension‐induced cardiac fibrosis and dysfunction are independently associated with increased morbidity and mortality risks [3, 4].

In response to increased afterload in hypertension, concentric hypertrophy occurs to maintain a normal systolic wall stress. Several studies demonstrated that hypertensive ventricular hypertrophy presented segmental heterogeneity, with the initial basal interventricular septum segment hypertrophy predominantly [5, 6, 7]. Moreover, this regional disparity was further evidenced by basal‐segment hypertrophy and systolic dysfunction [5]. The LV apical sparing pattern can reflect regional strain heterogeneity: the basal and mid segments are more severely impaired compared to the apical segment in cardiac amyloidosis [8]. In recent studies, this pattern has been found in hypertrophic obstructive cardiomyopathy [9] and severe aortic valve stenosis [10].

Although the global systolic function is maintained normally or even enhanced in hypertension, two‐dimensional speckle tracking echocardiography (2D‐STE) can effectively assess myocardial deformation and segmental contractile dysfunction [10]. Studies have shown that hypertension occurs with a normal left ventricular ejection fraction (LVEF), while global longitudinal strain (GLS) has decreased [5, 11, 12]. Segmental myocardial strain provides additional insight into localized myocardial injury or compensatory mechanisms [5, 13]. Compared to GLS, segmental longitudinal strain is more sensitive in evaluating the systolic dysfunction in hypertrophic cardiomyopathy and hypertension [5, 13].

There were limited investigations on the segmental heterogeneity of myocardial systolic function and myocardial fibrosis in hypertension. We aimed to investigate regional left ventricular deformation, regional myocardial fibrosis distribution, and the association of the apical sparing pattern with both structural remodeling and cardiac functional impairment in hypertension.

## 2 Methods

### 2.1 Ethical Approval

All procedures performed in studies were in accordance with the 1964 Helsinki Declaration. The study was approved by the Branch for Medical and Clinical Technology Application and Ethics Committee of the First Affiliated Hospital of Fujian Medical University [Approval No. MRCTA, ECFAH of FMU (2020) 396]. All procedures about animals were in compliance with the regulations and guidelines of Fujian Medical University's institutional animal care, and the study protocol was approved by the Ethics Committee of First Affiliated Hospital of Fujian Medical University (Approval ID: IACUC FJMU 2024‐Y‐1967).

### 2.2 Study Population

Seventy‐three essential hypertensive patients (aged 56.41 ± 11.19 years, 56 males and 17 females) who visited the First Affiliated Hospital of Fujian Medical University were enrolled in our retrospective study. The diagnosis of essential hypertension was made in accordance with the 2018 ESC/ESH Guidelines for the management of arterial hypertension [14]. A total of 23 healthy volunteers (aged 52.86 ± 7.77 years, 14 males and 9 females), who had no cardiovascular or systemic diseases, were recruited to serve as normal controls (NC). The following exclusion criteria were applied to the study cohort: poor image quality, secondary hypertension, established coronary artery disease (defined as coronary stenosis exceeding 50% on coronary angiography or a history of ischemic heart disease), significant valvular stenosis or regurgitation, congenital heart disease, atrial fibrillation, and idiopathic cardiomyopathy. According to the 2015 recommendations for cardiac chamber quantification by echocardiography in adults, left ventricular mass index (LVMI) > 115 g/m^2^ (male) or > 95 g/m^2^ (female) was defined as hypertrophy [15]. The enrolled hypertensive patients were divided into two groups: hypertension with normal LVMI (HT without LV hypertrophy, HT‐NLVH), hypertension with increased LVMI (HT with LV hypertrophy, HT‐LVH).

### 2.3 Experimental Animals

Six 16‐week‐old male spontaneously hypertensive rats (SHR) and six age‐matched male Wistar‐Kyoto (WKY) rats purchased from Charles River Laboratories (Beijing, China, SCXK2021‐0006) were maintained in the Laboratory Animal Center of Fuzhou Cold‐Spring Biology Company Limited (SYXK 2024‐0005) (SPF grade). All the animals were kept for 2 weeks in an environment with a temperature of 22°C ± 2°C and a 12 h/12 h light/dark cycle and fed with a standard diet before experiments.

### 2.4 Animal Blood Pressure Measurements

Systolic and diastolic blood pressure were determined through the tail‐cuff occlusion method (Coda Non‐Invasive Tail Blood Pressure System, SN:1234, Version: 210.10, CODATM Monitor, Kent Scientific Corporation) before the experiment. Each rat was individually coupled to the system, and each measurement was the average of three readings recorded.

### 2.5 Conventional Echocardiography for Animals

Routine echocardiography was performed to acquire left ventricular structural and functional parameters using GEVIVID E95 ultrasound equipment (GE Vingmed Ultrasound, Horten, Norway) with a 12S probe (frame rate > 200 FPS, 10.0–12.0 MHz). Rats were anesthetized and examined in the left lateral decubitus position. Electrocardiogram‐triggered echocardiographic views of the parasternal long‐ and short‐axis and apical 4‐chamber images were acquired and stored in a cine‐loop format for offline analysis. The following conventional parameters were measured according to the American Society of Echocardiography guidelines [15]: left atrial diameter (LAD), left ventricular internal dimension at end‐diastole (LVIDd) and end‐systole (LVIDs), interventricular septal thickness (IVST), left ventricular posterior wall thickness (LVPWT), peak mitral inflow velocity at early (PVE) and late (PVA) diastole, and the value of lateral and septal mitral annulus velocity at early diastole (e, e1). LVIDd and LVIDs were obtained through M‐mode tracing (at 100 mm/s) of the parasternal short‐axis view at the papillary muscle level. Fractional shortening (FS) was calculated by ([LVIDd−LVIDs]/LVIDd) × 100%. Relative wall thickness (RWT) and left ventricular mass (LVM)were then calculated [5, 16, 17]:RWT = (IVST + LVPWT)/LVIDd, LVM = [(LVIDd + IVST + LVPWT)^3^−LVIDd^3^] × 1.04. The left ventricular end‐diastolic volume (LVEDV) and left ventricular end‐systolic volume (LVESV) were calculated using Teichholz equations [18, 19]. LVEF was calculated as follows: LVEF = (LVEDV−LVESV)/LVEDV × 100%.

### 2.6 Conventional Echocardiography in Patients

Routine echocardiography was performed using GEVIVID E95 ultrasound equipment (GE Vingmed Ultrasound, Horten, Norway) with an M5s probe (Frame rate 50–80 FPS, 1.7–4.0 MHz). Transthoracic echocardiography was performed in the left lateral decubitus position at rest immediately after brachial blood pressure measurements were taken. Electrocardiogram‐triggered echocardiographic views of the parasternal long axis, apical 4‐chamber, apical 2‐chamber, and apical 3‐chamber images were acquired and stored in a cine‐loop format for offline analysis. The LVIDd, LVIDs, IVST, and LVPWT were measured at the parasternal long view. FS and LVM were calculated according to the 2015 ASE guideline [15]. The left atrial volume (LAV), PVE, PVA, e, e1 were measured at apical 4‐chamber view. LVEDV and LVESV were measured by the biplane Simpson method, and LVEF was calculated. The e‐average was the average value of septal and lateral mitral annulus velocity at early diastole (represented by e’). LAV, LVM, and LVEDV were adjusted for body surface area.

### 2.7 Two Dimensional‐Speckle Tracking Echocardiography (2D‐STE)

Left ventricular systolic strain measurements by 2D‐STE analysis were performed offline using EchoPAC Software (version 204, General Electric Company, Horten, Norway). The global and segmental myocardial strain analysis was performed by speckle tracking, and the displacement of speckles of myocardium was tracked frame by frame. After manually tracing the area of interest between the endocardial and epicardial borders, the software then automatically determined six segments in each view. The average of three levels of segments was defined as the segmental strain, including longitudinal strain of basal, middle, and apical segments (LS‐bas, LS‐mid, LS‐ap). Each segmental strain curve was obtained by frame‐by‐frame tracking of the acoustic markers in left ventricular myocardial tissue. The quality of every segmental tracking was scored as either acceptable or unacceptable. Longitudinal strain values for all six segments in each of the apical four‐chamber views were measured and averaged automatically to derive global longitudinal strain (GLS). For further understanding of the contribution of apical myocardial work to the global myocardial work, the following formulas were used to derive the proportion of the apical LS (P‐ap): Pap = LS‐ap/(LS‐ap + LS‐mid + LS‐bas), as described by Ding et al. [20].

### 2.8 Histological Indexes

After rat sacrifice, animal hearts were dissected and thoroughly washed with cold saline. The basal and apical segments of the left ventricle were cut carefully and immediately fixed in neutral formalin for Masson's trichrome staining, which was performed to observe cardiac fibrosis. The perivascular collagen area (PVCA) of basal and apical segments (PVCA‐bas, PVCA‐ap) was calculated as PVCA/vascular lumen area. Collagen volume fraction (CVF) of basal and apical segments (CVF‐bas, CVF‐ap) was calculated as myocardial collagen area/total myocardial tissue area × 100%. CVF and PVCA were analyzed and calculated by the Imagepro‐Plus 6.0 image analysis system.

### 2.9 Statistical Analyses

SPSS version 24 (IBM Corporation, Armonk, NY) was used to perform the statistical analysis. Normally distributed data were expressed as mean ± SD, and not normally distributed data are expressed as median with the 25th and 75th quartiles. Differences between groups were evaluated by the Student's *t*‐test or the non‐parametric test. ANOVA was used to assess multiple comparisons among groups. The interrelationship was explored by Spearman's correlation analysis. A value of *p* < 0.05 was considered to be statistically significant. Graphpad Prism 6.0 was used for all data graphing.

## 3 Results

### 3.1 Clinical Characteristics and Conventional Cardiac Structural and Functional Parameters in Patients

No inter‐group differences were observed in age, sex, height, weight, body surface area, or heart rate (all *p* > 0.05). Both hypertensive groups exhibited significantly higher systolic and diastolic blood pressures compared with the control group (*p* < 0.05). Compared with controls, the HT‐NLVH group showed progressive remodeling, evidenced by higher IVST, LVPWT, RWT, LVMI, and LVEDVI, which were further aggravated in the HT‐LVH group (all *p* < 0.05). Despite preserved systolic function (FS, LVEF), the HT‐NLVH group exhibited diastolic impairment (elevated LAVI, E/e’), which was more pronounced diastolic dysfunction (elevated LAVI, E/e’, PVA, and decreased e’) in the HT‐LVH group (*p* < 0.05) (Table 1).

**Table 1:** Clinical Characteristics and echocardiographic parameters in hypertensive patients with and without hypertrophy.

	NC (*n* = 23)	HT‐NLVH (*n* = 35)	HT‐LVH (*n* = 38)
Age (years)	52.86 ± 7.77	54.31 ± 10.62	58.34 ± 11.49
Male/female	14/9	28/7	28/10
Height (cm)	165.59 ± 6.67	167.09 ± 7.84	164.95 ± 9.42
Weight (kg)	64.07 ± 9.81	69.64 ± 11.22	67.95 ± 16.61
Body surface area (m^2^)	1.68 ± 0.16	1.76 ± 0.17	1.72 ± 0.26
Heart rate (beats/min)	70.20 ± 10.01	70.57 ± 11.45	67.11 ± 11.49
Systolic blood pressure (mm Hg)	121.23 ± 12.03	145.83 ± 18.86^a^	156.96 ± 21.70^a^
Diastolic blood pressure (mm Hg)	80.77 ± 8.30	91.30 ± 15.08^a^	93.44 ± 11.49^a^
LAVI (mL/m2)	22.84 ± 4.71	28.05 ± 7.25^a^	35.11 ± 10.52^a,b^
LVIDd (cm)	4.67 ± 0.25	5.12 ± 0.48^a^	5.30 ± 0.59^a^
IVST (cm)	0.88 ± 0.09	1.03 ± 0.15^a^	1.34 ± 0.31^a,b^
LVPWT (cm)	0.83 ± 0.10	0.89 ± 0.12	1.14 ± 0.21^a,b^
RWT	0.36 ± 0.03	0.38 ± 0.07	0.47 ± 0.11^a,b^
LVMI (g/m2)	78.87 ± 10.09	102.14 ± 13.76^a^	158.21 ± 41.13^a,b^
LVEDVI (mL/m2)	60.50 ± 5.27	72.30 ± 15.45^a^	79.78 ± 15.74^a,b^
FS (%)	34.83 ± 3.69	34.49 ± 4.72	34.52 ± 3.70
LVEF (%)	63.36 ± 3.52	61.13 ± 5.82	61.35 ± 4.25
PVA (m/s)	0.61 ± 0.14	0.70 ± 0.18	0.82 ± 0.24^a,b^
PVE (m/s)	0.67 ± 0.13	0.67 ± 0.18	0.64 ± 0.24
e' (m/s)	0.09 ± 0.02	0.08 ± 0.08	0.06 ± 0.02^a,b^
E/e'	7.46 ± 1.38	9.92 ± 3.81^a^	12.22 ± 4.38^a,b^
s (m/s)	0.08 ± 0.01	0.07 ± 0.02	0.07 ± 0.04
GLS (%)	−18.13 ± 2.68	−15.98 ± 3.01^a^	−14.14 ± 3.66^a^
LS‐bas (%)	−15.53 ± 3.19	−13.24 ± 3.00^a^	−10.46 ± 3.93^a,b^
LS‐mid (%)	−17.20 ± 2.94	−14.87 ± 2.89^a^	−12.98 ± 3.86^a,b^
LS‐ap (%)	−21.89 ± 3.70	−20.08 ± 4.88	−18.89 ± 5.37^a^
P‐ap	0.40 ± 0.04	0.42 ± 0.06	0.45 ± 0.09^a,b^

*Note:* ^a^ *p* < 0.05 versus NC group, ^b^ *p* < 0.05 versus the HT‐NLVH group.

Abbreviations: ‐ap, apical segments; ‐bas, basal segments; ‐mid, middle segments; e’, average early diastolic velocities of mitral annulus; FS, fractional shortening; GLS, global longitudinal strain; HT‐LVH, hypertension with left ventricular hypertrophy; HT‐NLVH, hypertension without left ventricular hypertrophy; IVST, interventricular septal thickness; LAVI, left atrial volume index; LS, longitudinal strain; LVEDVI, left ventricular end diastole volume index; LVEF, left ventricular ejection fraction; LVIDd, left ventricular internal dimension in diastole; LVMI, left ventricular mass index; LVPWT, left ventricular posterior wall thickness; NC, normal control; P‐ap (proportion‐ap), the proportion of LS‐ap/(LS‐bas + LS‐mid + LS‐ap); PVA, peak mitral orifice flow velocity at late diastole; PVE, peak mitral orifice flow velocity at early diastole; RWT, relative wall thickness; s, average peak systolic velocities of mitral annulus.

### 3.2 Global and Segmental Strain in Patients

**Figure 1:**
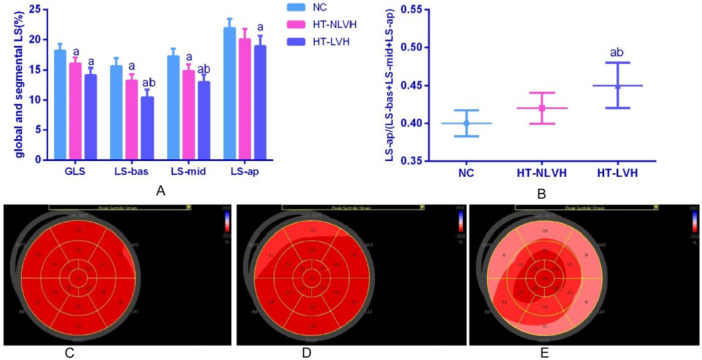
Global and segmental longitudinal strain and P‐ap in patients. ^a^ *p* < 0.05 versus NC group, ^b^ *p* < 0.05 versus HT‐NLVH group; (A) global and segmental longitudinal strain; (B) P‐ap; the bull's eye map of segmental myocardial of examples of patients from: (C) NC group; (D) HT‐NLVH group; (E) HT‐LVH group. ‐ap, apical segments; ‐bas, basal segments; GLS, global longitudinal strain; HT‐LVH, hypertension with left ventricular hypertrophy; HT‐NLVH, hypertension without left ventricular hypertrophy; LS, longitudinal strain; ‐mid, middle segments; NC, normal control; P‐ap(proportion‐ap), the proportion of LS‐ap/(LS‐bas + LS‐mid + LS‐ap).

Hypertensive groups exhibited impaired GLS versus controls (*p* < 0.05), with a trend toward worse values in HT‐LVH. Segmental analysis showed progressive dysfunction: LS‐ap declined only in HT‐LVH, while LS‐mid and LS‐bas demonstrated graded worsening from HT‐NLVH to HT‐LVH (*p* < 0.05). To better characterize the role of apical segmental strain in global myocardial function, we further analyzed P‐ap. The results demonstrated a modest but non‐significant increase in the HT‐NLVH group, while the HT‐LVH group exhibited a significantly elevated ratio, even when compared with the HT‐NLVH group (*p* < 0.05), as shown in Table 1, and Figure 1.

### 3.3 Correlation Analysis of P‐ap with Structural and Diastolic Function Measurements in Patients

Bivariate correlation analysis demonstrated a significant association between P‐ap and IVST, LVPWT, RWT, LVMI, and E/e’ (Table 2).

**Table 2:** Correlation analysis of P‐ap in patients.

		LAVI	IVST	LVPWT	RWT	LVMI	PVA	PVE	E/e'	e'
P‐ap	*r*	0.186	0.412	0.294	0.366	0.313	0.177	0.009	0.211	−0.082
	*p*	0.069	< 0.001	0.004	< 0.001	0.002	0.084	0.934	0.039	0.428

Abbreviations: IVST, interventricular septal thickness; LAVI, left atrial volume index; LVMI, left ventricular mass index; LVPWT, left ventricular posterior wall thickness; P‐ap (proportion‐ap), the proportion of LS‐ap/(LS‐bas + LS‐mid + LS‐ap); PVE, PVA, peak mitral orifice flow velocity at early diastole and late diastole; e’, average early diastolic velocities of mitral annulus; RWT, relative wall thickness.

### 3.4 Animal Characteristics and Conventional Echocardiographic Parameters

**Figure 2:**
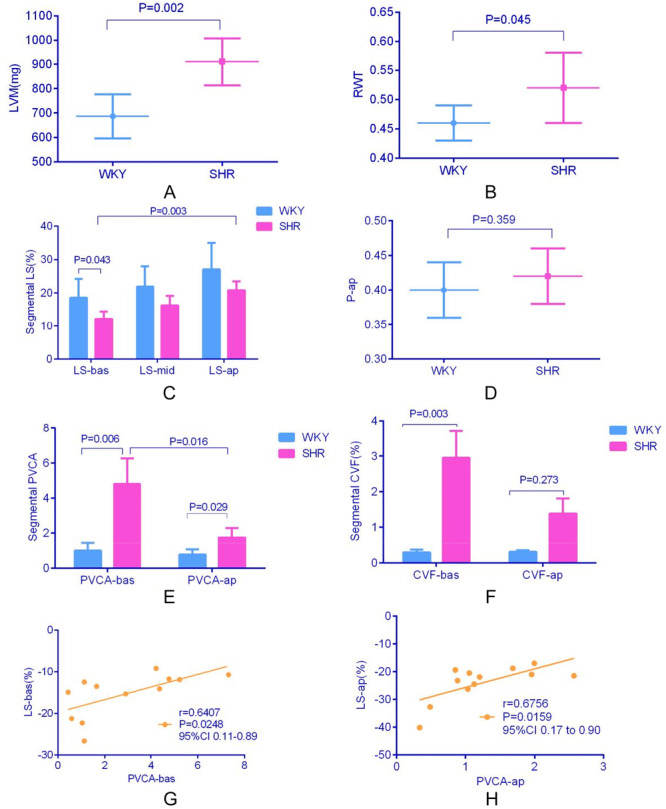
Animal left ventricular remodeling parameters (A, B), longitudinal strain (absolute value) (C), P‐ap (D), segmental PVCA (E), segmental CVF (F) and the correlation of PVCA and LS (G, H). ‐ap, apical segments; ‐bas, basal segments; CVF, collagen volume fraction; GLS, global longitudinal strain; LS, longitudinal strain; LVM, left ventricular mass; ‐mid, middle segments; P‐ap(proportion‐ap), the ratio of LS‐ap/(LS‐bas + LS‐mid + LS‐ap); PVCA, perivascular collagen area; RWT, relative wall thickness; SHR, spontaneously hypertensive rat; WKY, Wistar‐Kyoto.

Compared with WKY, SHR showed higher blood pressure (*p* < 0.05). There were no statistical differences for body weight, heart rate, LVIDd, LVIDs, LAD, and conventional systolic and diastolic function parameters between WKY and SHR (LVEF, FS, E, A, and e, e1). Indicators of hypertrophy and remodeling—including IVST, LVPWT, LVM, and RWT—were significantly elevated in the SHR group compared with controls (all *p* < 0.05), as shown in Table 3, Figure 2A,B.

**Table 3:** Animal characteristics and echocardiographic parameters.

	WKY (*n* = 6)	SHR (*n* = 6)	*p* value
Body weight (g)	286.93 ± 9.85	297.00 ± 10.30	0.114
Heart rate (beats/min)	368.67 ± 38.78	311.83 ± 81.44	0.166
Systolic blood pressure (mm Hg)	119.42 (112.00−126.85)	204.57 (183.93−204.88)	0.004
Diastolic blood pressure (mm Hg)	83.80 ± 5.96	142.35 ± 13.58	< 0.001
LAD (mm)	4.57 ± 0.15	4.68 ± 0.15	0.270
LVIDd (mm)	6.80 ± 0.47	7.07 ± 0.57	0.402
LVIDs (mm)	3.71 ± 0.25	3.82 ± 0.30	0.528
IVST (mm)	1.58 ± 0.09	1.97 ± 0.04	< 0.001
LVPWT (mm)	1.52 ± 0.03	1.67 ± 0.06	< 0.001
RWT	0.46 ± 0.03	0.52 ± 0.06	0.045
LVM (mm)	686.56 ± 90.91	910.94 ± 97.01	0.002
LVEDV (uL)	240.52 ± 36.48	262.68 ± 45.03	0.371
FS (%)	46.70 (45.63−47.77)	43.98 (43.97−45.26)	0.423
LVEF (%)	75.45 ± 1.52	75.92 ± 1.73	0.632
PVE (m/s)	1.21 ± 0.12	1.15 ± 0.26	0.652
PVA (m/s)	0.57 ± 0.15	0.52 ± 0.26	0.808
e (mm/s)	52.91 ± 10.45	44.68 ± 11.79	0.229
e1 (mm/s)	57.27 ± 16.36	50.26 ± 11.64	0.412
GLS (%)	−22.49 ± 6.19	−16.39 ± 2.05	0.062

Abbreviations: e, e1, early diastolic velocities of mitral annulus; FS, fractional shortening; GLS, global longitudinal strain; LAD, left atrial diameter; LVEDV, left ventricular end diastole volume; LVEF, left ventricular ejection fraction; LVIDd, LVIDs, left ventricular internal dimension in diastole and end‐systole; LVM, left ventricular mass; LVPWT, left ventricular posterior wall thickness; PVE, PVA, peak mitral orifice flow velocity at early diastole and late diastole; RWT, relative wall thickness.

### 3.5 Global and Segmental Longitudinal Strain in WKY and SHR

The SHR group exhibited a reduction in GLS, though this did not reach statistical significance (*p* = 0.062, Table 3). For segmental longitudinal strain, it showed an increasing trend from basal to apical segments in WKY and SHR rats. Compared with WKY, LS‐bas was reduced significantly in SHR (*p* = 0.043). LS‐mid and LS‐ap showed a decreasing trend, but still did not reach a statistically different level (Figure 2C), as well as GLS. The P‐ap showed an increased trend in SHR, as shown in Figure 2D.

### 3.6 Expression of Myocardial Collagen in Different Segments

**Figure 3:**
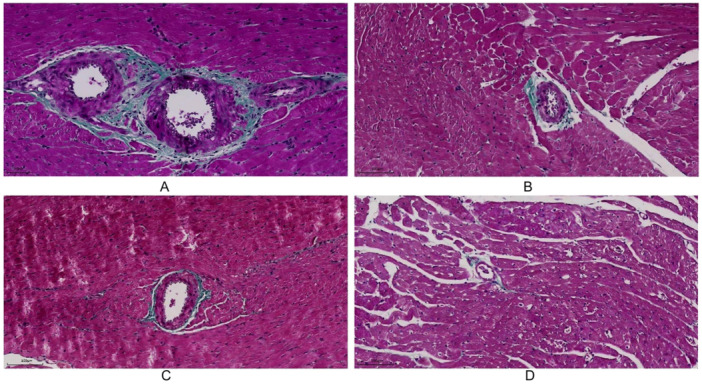
Segmental PVCA in WKY and SHR. (A) PVCA‐bas in SHR; (B) PVCA‐ap in SHR; (C) PVCA‐bas in WKY; (D) PVCA‐ap in WKY; ‐ap, apical segments; ‐bas, basal segments; PVCA, perivascular collagen area; SHR, spontaneously hypertensive rat; WKY, Wistar‐Kyoto.

The SHR group demonstrated significantly elevated PVCA and CVF compared with WKY controls, with particularly pronounced accumulation in the basal region (PVCA‐bas, CVF‐bas) that achieved statistical significance (PVCA‐bas: 4.80 ± 1.46 vs. 1.00 ± 0.44, *p* = 0.006; CVF‐bas: 2.95 ± 1.85% vs. 0.29 ± 0.19%, *p* = 0.003). PVCA‐ap increased significantly in the SHR group (1.76 ± 0.54 vs. 0.78 ± 0.30, *p* = 0.029), whereas CVF‐ap showed no statistically significant differences between groups (CVF‐ap: 1.39 ± 1.02% vs. 0.31 ± 0.10%, *p* = 0.273), Figure 2E,F and 3).

### 3.7 Correlation Analysis of Segmental LS Parameters with PVCA

Bivariate correlation analysis demonstrated a significant association between LS and PVCA in basal and apical segments (Figure 2G,H).

### 3.8 Intra‐ and Inter‐Observer Reproducibility Assessment

Intraclass correlation coefficients (ICCs) were calculated for interobserver and intra‐observer agreement in five randomly selected rats. Myocardial strain parameters had exhibited good intra‐ and inter‐observer correlation in our previous work [21, 22]. PVCA parameters were re‐measured by two independent observers who were blinded to all other animal data and each other's results for interobserver productivity. The intra‐observer reproducibility was achieved at 1‐week intervals between the first and second measurements by the same observer (Table 4).

**Table 4:** Interclass correlation coefficient for intra‐ and inter‐observer variability for myocardial PVCA parameters.

	Inter‐observer variability			Intra‐observer variability		
	ICC	95% CI	*p* value	ICC	95% CI	*p* value
PVCA‐bas	0.963	0.693−0.996	0.001	0.992	0.929−0.999	< 0.001
PVCA‐ap	0.953	0.626−0.995	0.002	0.941	0.548−0.994	0.003

Abbreviations: CI, confidence interval; ICC, intraclass correlation coeffificients; PVCA‐bas, perivascular collagen area of basal segments; PVCA‐bas, perivascular collagen area of apical segments.

## 4 Discussion

In the present study, the segmental myocardial strain was a sensitive and accurate index to detect the systolic dysfunction in hypertensive individuals and SHR. The principal findings were as follows: (1) In the clinical study, segmental strain analysis revealed progressive systolic dysfunction: LS‐basal and LS‐mid were first reduced in the HT‐NLVH group, followed by apical involvement, and the LS‐basal and LS‐mid deteriorated further, accompanied by an increased apical preservation ratio (P‐ap). In addition, P‐ap correlated with hypertrophy markers (LVMI, IVST, LVPWT, and RWT) and diastolic function (E/e′). (2) Fibrosis displayed regional heterogeneity: the basal segments showed significant collagen deposition, whereas apical segments exhibited selective perivascular fibrosis without diffuse interstitial changes, paralleling strain dysfunction.

### 4.1 Hypertrophy and Segmental Dysfunction: Early Markers of Hypertensive Heart Disease

Though in the HT‐NLVH group, the individuals presented normal LV morphology according to the recommendation, the LVMI and IVST increased compared to the NC, which was considered the early stage in hypertensive remodeling [11, 13]. In the HT‐NLVH group and SHR group, the LS‐basal decreased first while the LVEF was preserved. Previous study showed that in mild to moderate hypertension, the hypertrophy remodeling disproportionately occurred, which was first presented in the basal segmental and the segmental SL decreased in parallel [5]. From infancy to childhood, the left ventricular diameter and ventricular mass showed progressive growth with age [23]. In normal subjects, left ventricular wall thickness exhibits a characteristic base‐to‐apex decrement pattern, and the LV chamber presents a conical shape [5]. Based on the Laplace law, the wall stress is dependent on local LV geometry. The radius of curvature at the cardiac apex was significantly smaller compared to both the lateral wall and septal radius of curvature at the basal ventricular level, which both induced higher wall stress in the basal segment [24]. And with the rise of blood pressure, stress increased disproportionately. The imbalance increased wall stress, triggered segmental hypertrophy, and myocardial dysfunction. As the progression of LVH, the basal and middle LS decreased more severely than the apical LS in our study. Consequently, wall stress‐associated myocardial injury manifests initially in the basal segmental and later and milder at the apex. The basal segmental SL was a reliable and sensitive parameter for detecting early damage in hypertension.

### 4.2 Focal Myocardial Dysfunction: Linking Fibrosis Patterns

In this study, we found that in SHRs compared to WKY rats, the distribution of both PVCA and CVF increased at the basal segments. At the apical segment, PVCA increased, whereas CVF did not change, a pattern similar to that reported in another study [25]. The distribution of PVCA and CVF was significantly higher in the basal than apical segment in the SHR group. The elevated myocardial wall stress and rennin‐angiotensin‐aldosterone system play an important role in myocardial remodeling and interstitial and perivascular fibrosis [26]. The LV hypertrophy and the myocardial fibrosis first happened at the basal segment, and the PVCA accelerated progressive early and higher than CVF [27], which was also found in our study. According to Laplace's law, the distribution of elevated wall stress in basal myocardial segments may initiate a cascade of inflammatory signaling and cellular responses that trigger regional progressive fibrosis [28].

The GLS was associated with myocardial fibrosis and considered a remarkable index for fibrosis in previous study [22]. While in the patchy distribution of fibrosis disease, the segmental LS was more precise and sufficient than GLS [29]. In this study, we found that the segmental LS was associated with the segmental fibrosis. The basal and mid‐septal segments exhibit the most pronounced LS impairment in response to hypertrophic/fibrotic remodeling with increased afterload [25]. The hypertrophy and the collagen deposition in the perivascular tissues play an important role in decreasing coronary flow reserve and impaired vasodilator reserve [30], which may consequently lead to myocardial ischemia and systolic dysfunction.

### 4.3 The Relative Apical Sparing Pattern and Cardiac Function in Hypertension

In hypertension, despite decreased GLS and progressively more pronounced relative apical sparing with advancing hypertrophy, the global systolic function(LVEF and FS) was preserved, which was consistent with previous studies [11, 12]. The LS gradient from base to apex results in a higher ejection fraction at the apex than at the base [24]. Apical rotation drives left ventricular twist via apex‐base counter‐rotation, creating a wringing motion that stores energy for powerful ejection [31]. Enhanced LV twist in hypertensive patients during the stages of concentric remodeling and hypertrophy contributed to maintaining a normal LVEF [32]. Recent studies showed that the relative apical sparing pattern indicated preserved apical contractility may serve as a compensatory mechanism for the reduced basal and mid‐ventricular strain, thereby maintaining a normal LVEF [11, 12].

In this study, our findings demonstrated that P‐ap progressively increased with the advancement of LV remodeling, hypertrophy, and diastolic dysfunction. This elevation in P‐ap reflected both the impairment of basal/mid‐ventricular strain and the relative apical strain sparing. Elevated P‐ap values paralleled more severe basal segment impairment, evidenced by significantly reduced regional strain and increased fibrosis deposition in these segments. Furthermore, our correlation analysis revealed significant associations between P‐ap and established markers of LV hypertrophy/remodeling, E/e′ ratio, and PVCA, reinforcing that this parameter not only captured the role of apical sparing in preserving systolic function but also suggested its potential as an indicator for tracking the progression of LV hypertrophic remodeling and diastolic deterioration.

Collectively, the heterogeneous fibrosis distribution between apical and basal segments induces early global diastolic dysfunction and segmental myocardial strain impairment while maintaining preserved global systolic contraction. This differential remodeling pattern, coupled with the apex's relatively reduced fibrotic involvement, underlies the characteristic apical sparing phenomenon in hypertension. Importantly, this spatial strain pattern may serve as a potential biomarker reflecting both diastolic impairment and hypertrophic progression.

### 4.4 Limitations

(1) Elevated heart rates in the rats resulted in fused E/A wave forms, a known physiological constraint in small animal models. Future investigations should incorporate heart rate modulation techniques to ensure accurate diastolic parameter assessment. (2) The present study results are derived from a small sample size, so a larger sample size is needed in the future. (3) The current analysis did not incorporate antihypertensive treatment information, as medication adherence data were either inconsistent or unavailable for some patients in the study cohort.

## 5 Conclusion

Early hypertensive remodeling is characterized by basal‐predominant fibrosis and strain impairment with relative apical sparing. With progression of hypertrophy and diastolic dysfunction, apical sparing becomes increasingly prominent. This spatial pattern may not only contribute to preserved global systolic function but could also potentially serve as a clinically observable characteristic feature, possibly reflecting the severity of diastolic impairment and hypertrophic remodeling progression.

## Author Contributions

All authors contributed substantially to the study's conception, design, and data interpretation. Huimei Huang, Chunyan Huang, and Yongxin Wu drafted the initial manuscript. Huimei Huang supervised data validation and resource acquisition. Meiyan Lin, Yupeng Chen, and Shengnan Lin conducted formal data analyses and methodological optimization. Liyun Fu and Huimei Huang provided senior oversight, contributing to analytical frameworks and study design. All authors critically revised the manuscript and approved the final version.

## Consent

The authors have nothing to report.

## Conflicts of Interest

The authors declare no conflicts of interest.

## Data Availability

The data that support the findings of this study are available from the corresponding author upon reasonable request.
